# Supplementary material for: Continuous transcription initiation guarantees robust repair of all transcribed genes and regulatory regions
Source: Nat Commun. 2020 Feb 14;11:916. doi: 10.1038/s41467-020-14566-9 (PMC7021815; doi:10.1038/s41467-020-14566-9)
Supplement: Supplementary file 1 — Supplementary Information [file 41467_2020_14566_MOESM1_ESM.pdf]

Continuous transcription initiation guarantees robust repair of all transcribed genes and regulatory regions

Liakos *et al.*

Supplementary Information

(Supplementary Figures + Supplementary Methods)

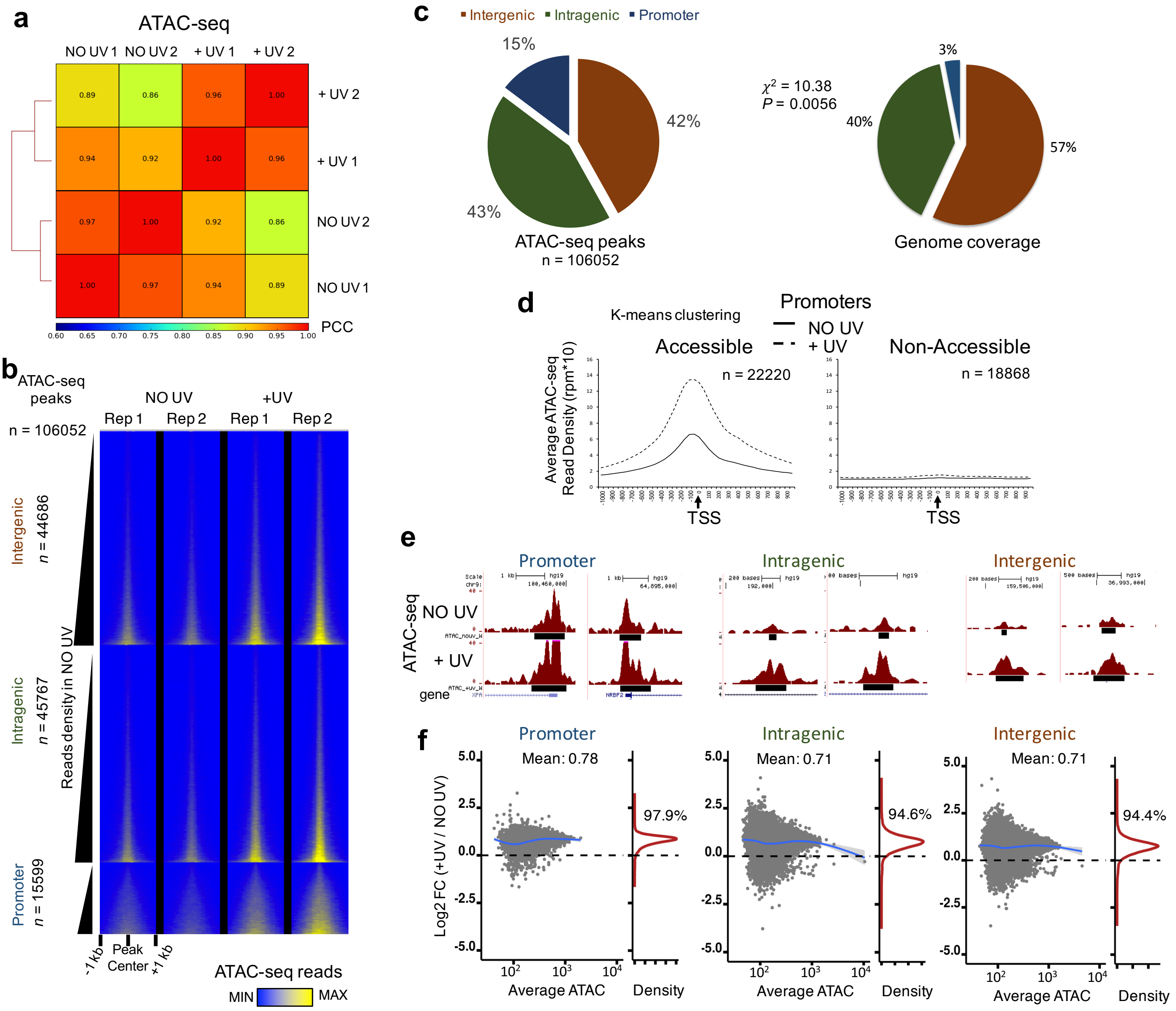

Supplementary Figure 1. Increase of chromatin accessibility in response to UV irradiation. **a** Correlation plot of ATAC-seq read densities for biological duplicates of non-irradiated (NO UV 1,2) and irradiated (+UV 1, 2; 15 J/m^2, +2 h) cells. Pearson's Correlation Coefficients (PCC) were calculated genome wide (3 kb windows) and reported on the heatmap. See Methods for details. **b** Heatmap depicting ATAC-seq peaks of two biological replicates separately (Rep 1, Rep 2) for NO UV and +UV condition in promoter, intragenic and intergenic regions as described in Fig. 1b for the union of peaks detected in NO UV and + UV conditions. Peaks are sorted by increasing read density in the merged NO UV. **c** Distribution of ATAC-seq peaks in promoter (+/-2kb relative to TSS), intragenic and intergenic regions (left part), compared to the genome percentage covered by the respective regions (right part). Chi-square test were performed to determine whether observed values differ from expected value purely by chance. **d** Average plots depicting ATAC-seq read densities in k-means resolved clusters of accessible and non-accessible promoters for non-irradiated (NO UV, solid line) and irradiated (+UV, dashed line) cells. **e** UCSC snapshot of ATAC-seq profiles of representative loci showing signal profile before (upper part) and after (lower part) UV, in promoter, intragenic and intergenic regions. **f** MA plots showing the individual (grey dots) and average (blue line) FC (Log_2 FC) in ATAC read density at ATAC-seq peaks, between +UV and NO UV, as a function of the average (from replicates) ATAC-seq read density in NO UV. Percentage of peaks with increased FC (Log_2 FC > 0) is indicated on the kernel density plot on the right of each MA plot

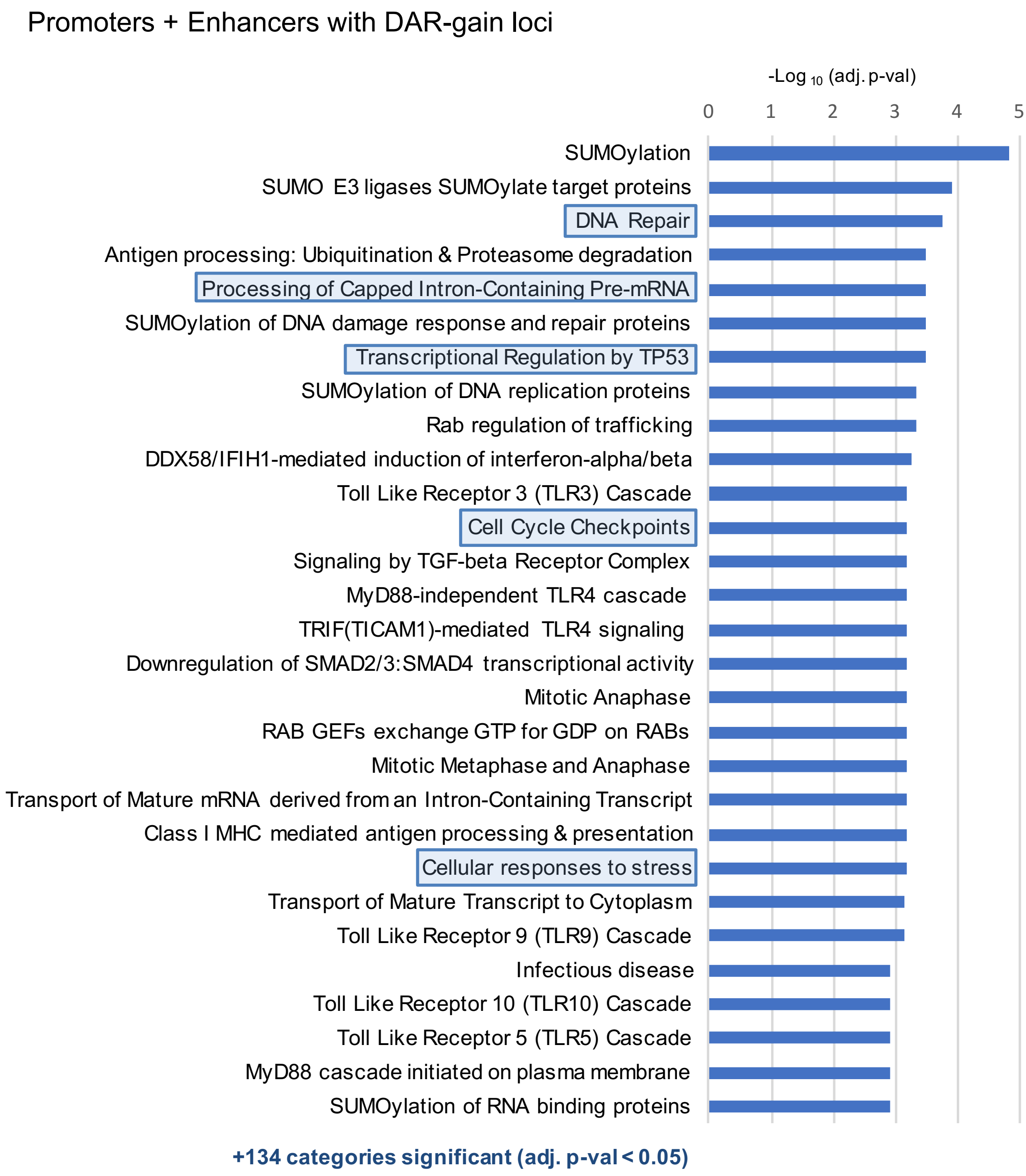

Supplementary Figure 2. Gene Ontology analysis of DAR-gain regions. Reactome biological pathway analysis (see Methods) for genes that are associated with DAR-gain genomic regions (either promoter-proximally associated with DAR-gain loci or controlled by at least one intergenic/intragenic DAR-gain region annotated as enhancer and associated topologically to a given gene according to FANTOM5 consortium). Pathways are sorted by decreasing significance. Highlighted biological pathways are relevant to the processes involved in UV-response or transcription

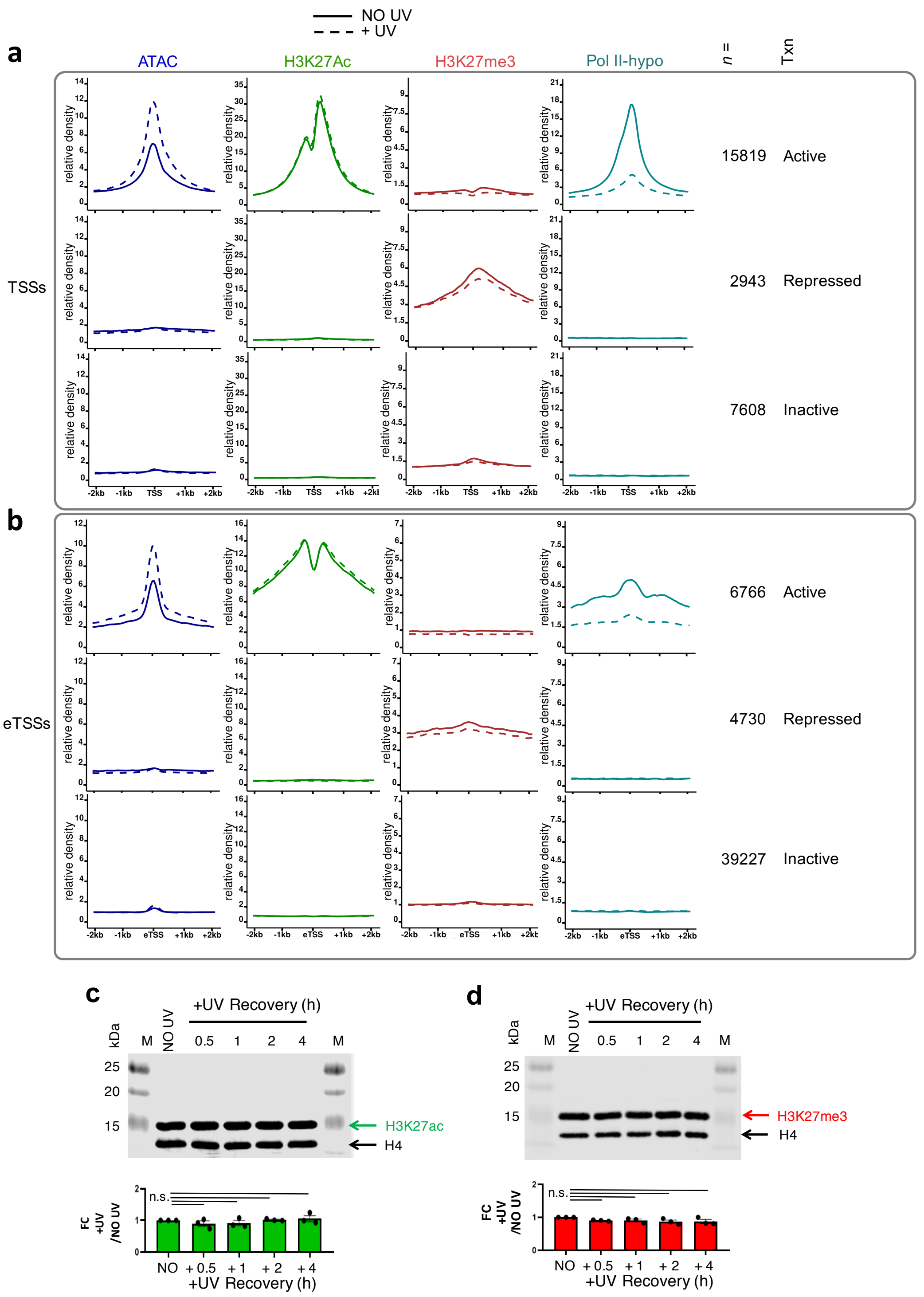

Supplementary Figure 3. Chromatin modifications remain virtually stable upon UV damage. a,b Average profile plots illustrating the read densities for ATAC-seq, H3K27ac, H3K27me3 and Pol II-hypo ChIP-seq before (NO UV, solid line) and after UV (+UV, dashed line), on active, inactive and repressed TSSs **(a)** and eTSSs **(b)** clusters defined in Fig. 2. (Transcription levels of the corresponding genes indicated in Txn column) **c** Western Blot analysis of histone extracts for bulk H3K27ac levels before and after different times during early recovery period (0.5-4 h) from UV irradiation. Histone 4 (H4) was used as a loading control. Quantification was calculated and compared to NO UV condition. Ordinary one-way ANOVA Dunnett's multiple comparisons test non-significant (n.s) between NO UV and +UV conditions. The figure is representative of 3 independent experiments. **d** Same as in **(c)** but for H3K27me3

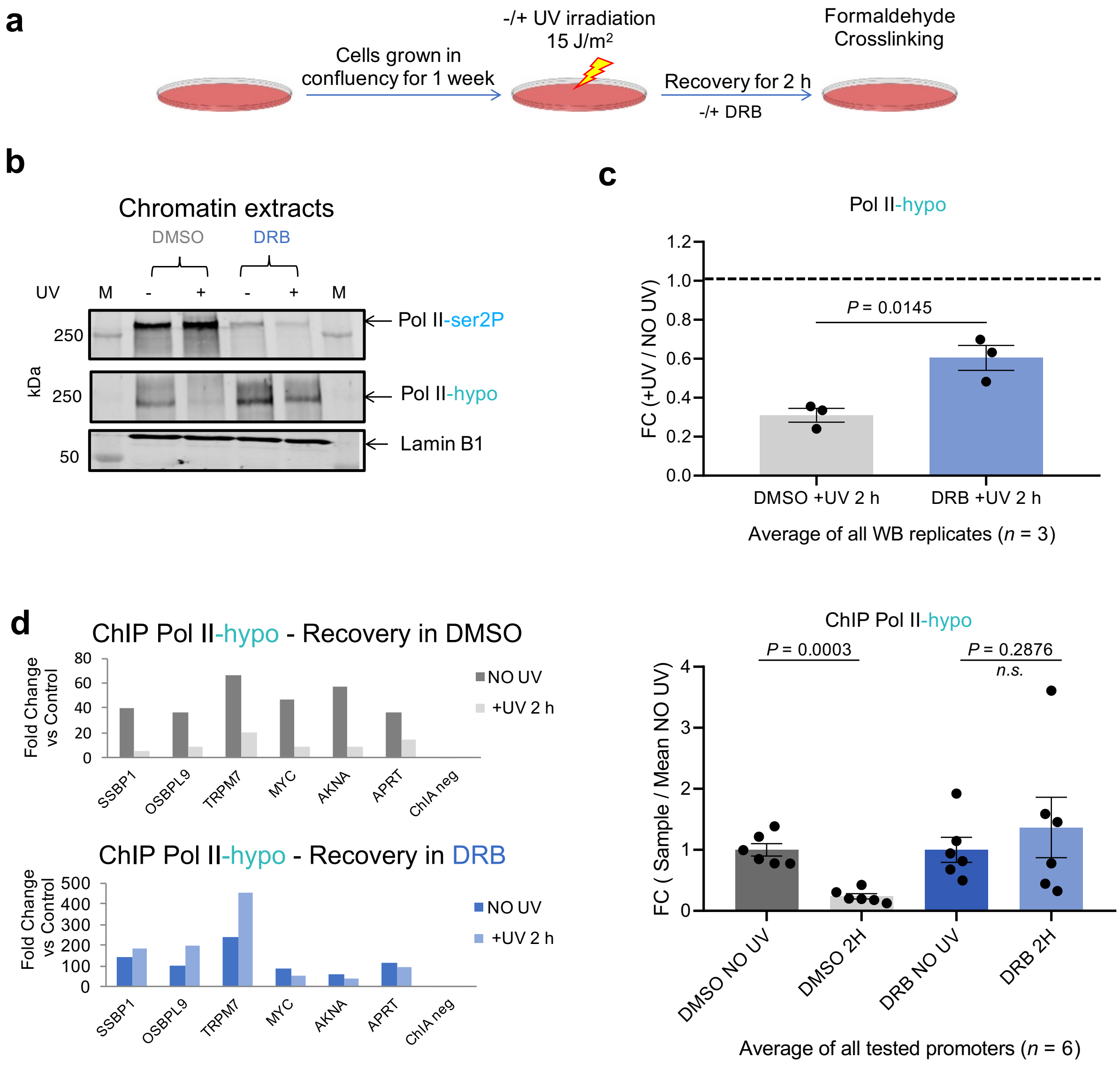

Supplementary Figure 4. Rescuing levels of pre-initiating Pol II (Pol II-hypo) after UV irradiation. a Experimental timeline. DRB was added in a final concentration of 100 μM. **b** Western Blot analysis of chromatin extracts for Pol II-ser2P and Pol II-hypo in the experimental conditions outlined in **(a)**. Lamin B1 was used as loading control. The plot is representative of 3 independent experiments. **c** Quantification of the average signal detected in **(b)** as compared to NO UV (dashed line) condition for DMSO and DRB treated cells, respectively. Error bars represent S.E.M. and *P* values are calculated using two-sided Student's t test. **d** (Left) ChIP-qPCR analysis of Pol II-hypo enrichment at the promoter regions of 6 representative genes selected from **Fig. 3** for cells treated as indicated in (a) with DMSO or with DRB. FC was calculated against a negative locus (ChIA neg). (Right) Average ChIP-qPCR enrichment of all genes tested (error bars represent +/- SEM). Error bars represent S.E.M. and *P* values are calculated using two-sided Student's t test

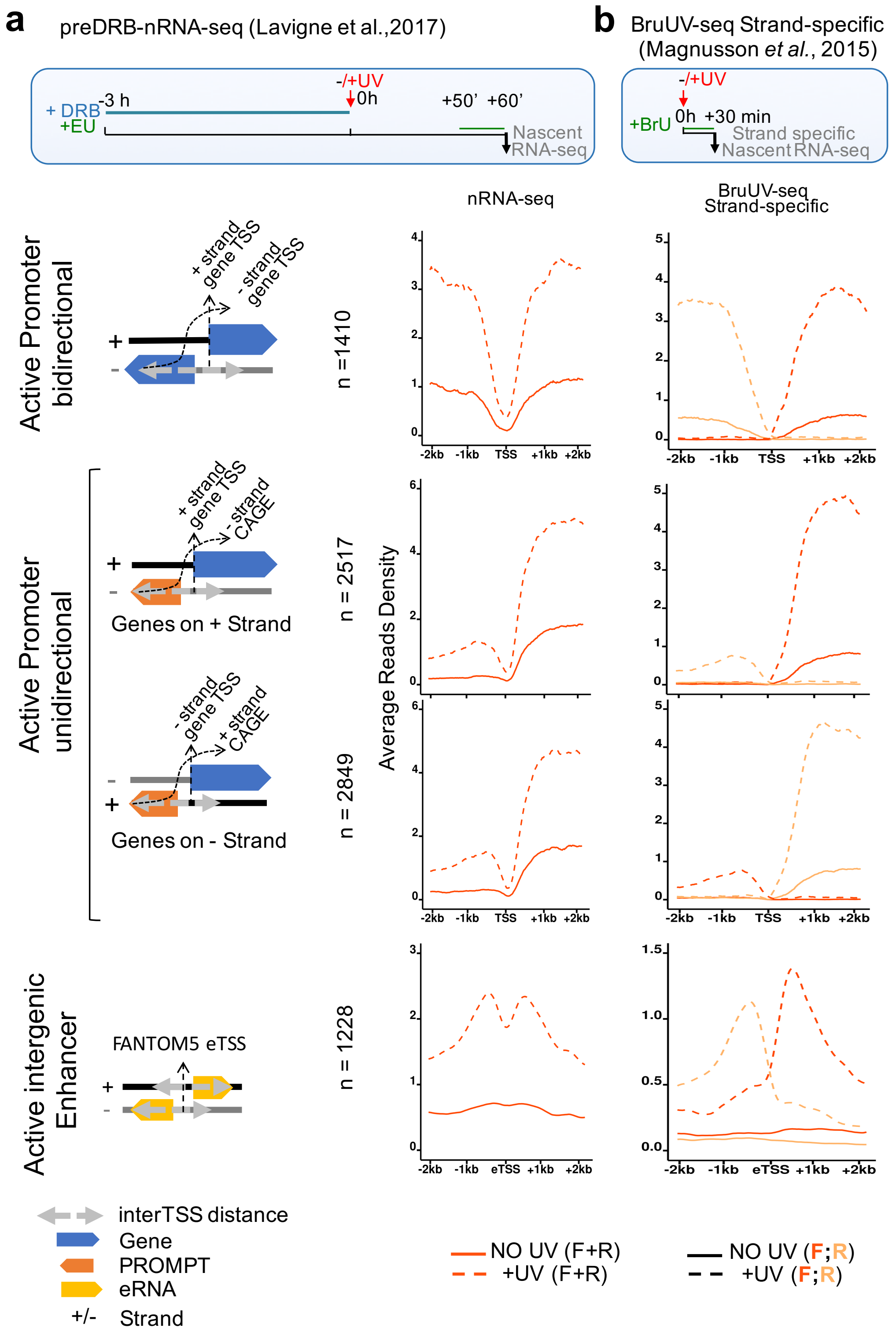

Supplementary Figure 5. *De novo* and increased RNA synthesis from all TSSs upon UV exposure. a (Upper part) Scheme for preDRB-nRNA-seq methodology (see[1]). Data used were the same described in Fig. 5. (Lower part) Average Read densities profiles of nRNA-seq, before (solid line) and after UV (dashed line) in genomic regions 2kb for categories defined in Fig. 3a. **b** Same as in **(a)** for strand-specific protocol of BruUV-seq. See Methods for details

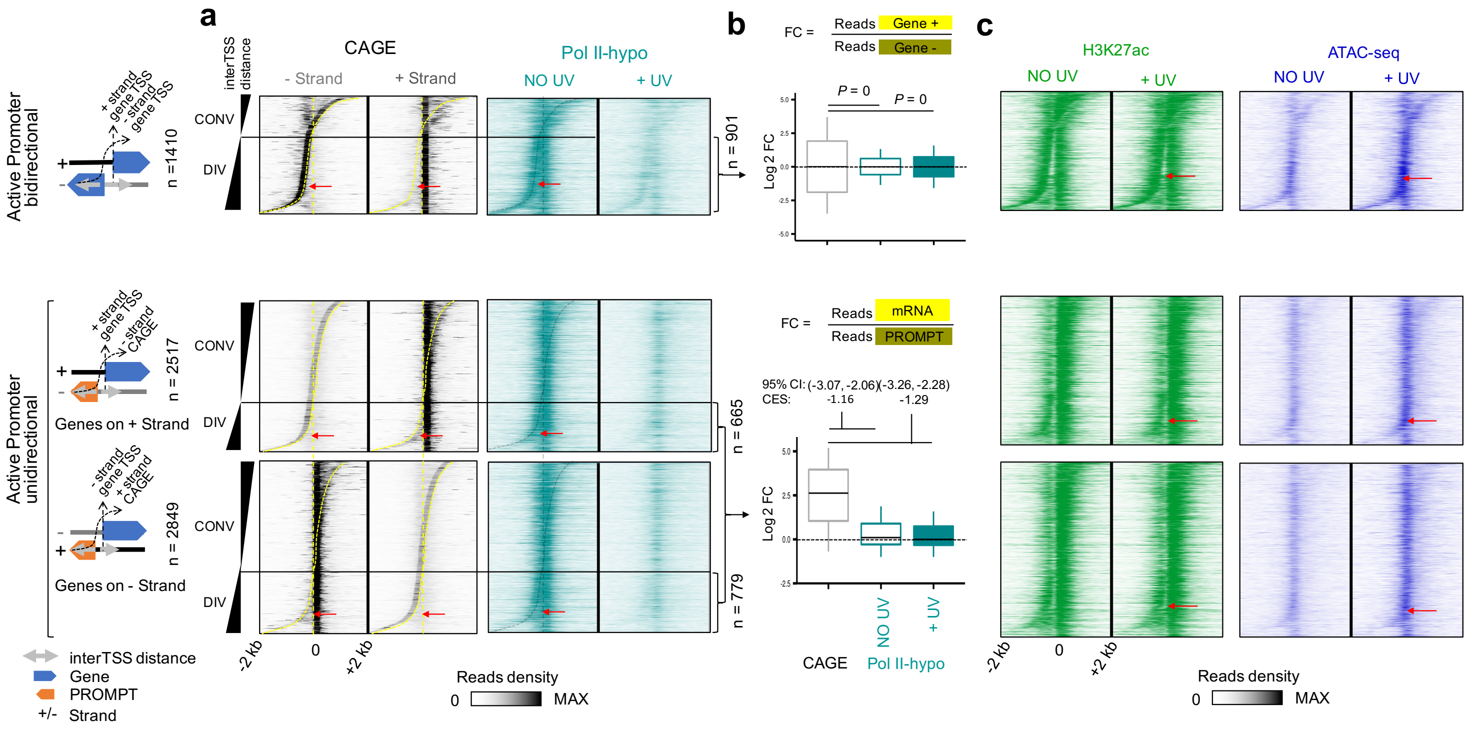

Supplementary Figure 6. Equal recruitment of Pol II on the flanks of NDR primes for increased transcription initiation from gene TSS and divergent PROMPTs upon UV. a Heatmaps of read densities of CAGE (- and + strand) and Pol II-hypo ChIP-seq (Data for Pol II-hypo are obtained from[1] and for CAGE from[2], see Methods) at steady state condition (NO UV) and upon UV (+1.5 h, 8 J/m^2), in genomic regions extending 2 kb around TSSs for categories defined in Fig. 3a**. b** Box plots showing quantifications for CAGE and Pol II-hypo ChIP-seq reads showed in **(a)** of the ratio between (+ strand) over (- strand) for divergent bidirectional promoters (upper) and mRNA over PROMPT (lower) for divergent unidirectional promoters. Box plots show the 25th–75th percentiles, and error bars depict data range to the larger/ smaller value no further than 1.5 * IQR (inter-quartile range, or distance between the first and third quartiles). (Upper) Two sample F-tests were conducted for each of 10,000 sampling pairs of 100 data points with replacement from each population, to test for significant difference between sample variance. The calculated *P* expresses the percentage of the non-significant F-tests (F-test p-value >= 0.05) out of the 10,000 total tests. (Bottom) 95 % confidence intervals (CI) of mean differences between log$_2$ counts of tested conditions were calculated for 10,000 samplings of 100 data points with replacement from each population. Effect sizes of log$_2$ counts between datasets were calculated using Cohen's method (CES). **c** Same as in **(a)** for H3K27ac ChIP-seq (green) and ATAC-seq (blue) reads before and after UV (+2 h, 15 J/m^2). Red arrows indicate the Nucleosome Depleted Regions (NDRs) for active divergent bidirectional and unidirectional promoters

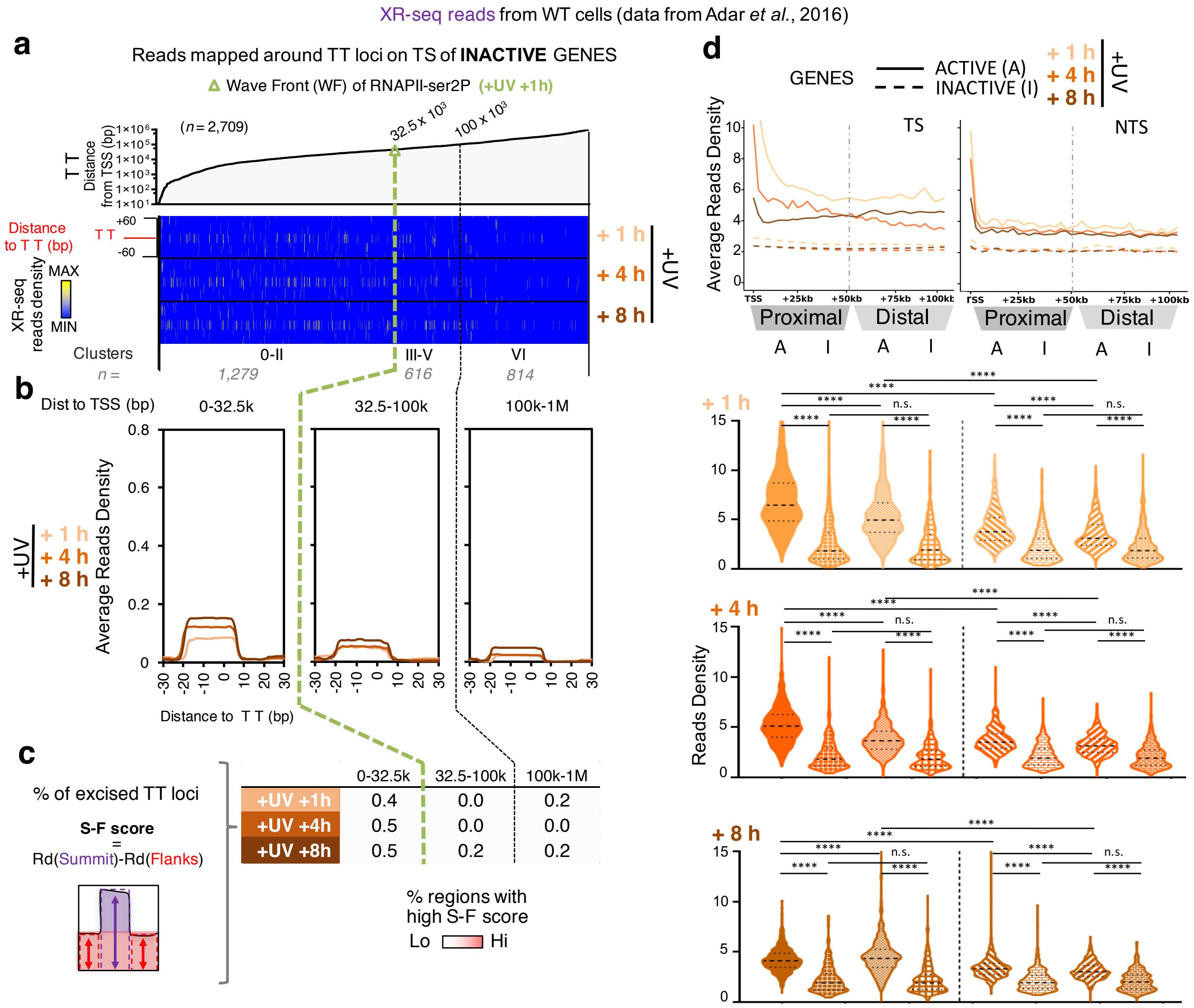

Supplementary Figure 7. Continuous initiation maintains TC-NER efficiency over time and across the whole transcribed region of active genes, regardless of their length. a Distribution of XR-seq reads derived from WT cells at 1h, 4h and 8h post UV irradiation. Reads were aligned on TT loci of transcribed strand (TS) of inactive genes. Data were the same as the one described in Fig. 8. Pol II-ser2P wave front position (WF =32.5 kb) was defined at +UV (+1 h) in[1]. **b** Average plots of read densities showed in **a** for the respective clusters **c** Plot showing the percentage (%) of excised TT loci, as calculated by the difference between Rd at summit (S) and Rd at flanks(F) (S-F score) from all clusters presented and analysed in **a** and **b**. (**a-c** should be compared with active genes data shown in Fig. 7) **d** (Upper) Average plot depicting reads density of XR-seq signal in proximal (from TSS to +50 kb) and distal (+50 kb to +100 kb) genomic regions of transcribed and non-transcribed strand (TS and NTS, respectively), in active (solid line, A) and inactive (dashed line, I) genes, respectively. (Lower) Violin plots showing the distribution of reads density of XR-seq detected in the average profile above. Thin dashed lines show the 25th–75th percentiles, and bold dash lines depict median value for the dataset. n.s. indicate non-significant P-value (> 0.99), **** indicate P-value < 0.0001 (Kruskall-Wallis non-parametric test using *ad hoc* pairwise Dunn's multiple comparison test)

Supplementary Methods

ChIP-seq

Formaldehyde was added to the cells at a final concentration of 1% at 4°C for 12 mins. The reaction was quenched by adding glycine (final concentration of 125 mM) for 5 mins. Cells were washed 3 times with cold PBS and then collected in PBS containing 1 mM EDTA, 0.5 mM EGTA and 1 mM PMSF and pelleted in pellets of ~$2x10^7$ cross-linked cells. Pellets were resuspended in Chro-IP lysis Buffer (50 mM Hepes-KOH pH 8.0, 1 mM EDTA, 0.5 mM EGTA, 140 mM NaCl, 10 % glycerol, 0.5 % IGEPAL, 0.25 % Triton X-100, 1 mM PMSF, and a mix of protease inhibitors (Roche)). After 10 min rotation at 4°C, cell suspension was centrifuged (10 min, 750 rcf at 4°C) and supernatant was kept as soluble fraction. In turn, cell pellet was washed with Wash Buffer (10 mM Tris-HCl, pH 8.0, 1 mM EDTA, 0.5 mM EGTA, 200 mM NaCl, 1 mM PMSF, 10 mM NaPy and protease inhibitors). The cell suspension was rotated for 10 min at 4°C and centrifuged as mentioned above. The cell pellet was resuspended in RIPA Buffer (10 mM Tris-HCl, pH 8.0, 1 mM EDTA, 0.5 mM EGTA, 140 mM NaCl, 1% Triton X-100, 0.1% Na-Deoxycholate, 0.1% SDS, 1 mM PMSF, 10 mM NaPy and protease inhibitors). Samples were sonicated using the Bioruptor water bath sonicator (Diagenode) using the "high" setting with cycles of 30 sec "on" and 30 sec "off", for a total duration of 25 minutes. Samples were then centrifuged for 10 min at 9500 rcf at 4°C. The supernatant was kept as chromatin fraction (Input). Chromatin immunoprecipitation (ChIP) was performed by incubation of equal amounts of sheared chromatin from irradiated and non-irradiated cells with the appropriate antibody at 4°C overnight.

The antibodies used for ChIP were the following: H3K27ac (ab4729, Abcam), H3K27me3 (07-449, Millipore), 8WG16 (Pol II-hypo) (05-952, Millipore). Protein A (for H3K27ac and H3K27me3) or Protein G (for 8WG16) Dynabeads (ThermoFisher Scientific) were blocked overnight at 4°C in RIPA buffer without protease inhibitors, NaPy and PMSF in the presence of Bovine Serum Albumin (BSA) (30µg/ml). Next day, beads and immunoprecipitated chromatin were co-incubated for 3 h at 4°C and then beads were sequentially washed twice with RIPA, three times with RIPA containing 0.3M NaCl, once with LiCl buffer (0,25M LiCl, 10mM Tris-HCl PH 8.0, 1mM EDTA, 0.5mM EGTA, 0.5% Triton X-100, 0.5% Sodium deoxycholate) and twice with TE buffer (10 mM Tris-HCl PH 8.0, 1 mM EDTA pH 8.0). Chromatin immuno-complexes were eluted by two rounds of incubation at 65°C for 20min in 1% SDS and 100 mM NaHCO3, and vigorous vortexing. Finally, de-crosslinking of Input and immunoprecipitated chromatin was performed by overnight incubation at 65°C in the presence of 200 mM of NACl. We then applied Proteinase K treatment (0.1µg/µl in 0.5% SDS) for 1h at 55°C, and DNA purification was performed with AMPURE XP Beads (Agencourt) according to manufacturer's protocol.

Histone acetic extraction

Cells grown to confluence in 10 cm plates, synchronized and released as described above, were irradiated or not with 15 J/m^2 and treated cells were left to recover for indicated time points before harvesting. Cells were washed 3 times with cold PBS, harvested and centrifuged at 380 rcf for 5 min at different recovery periods as indicated in Supplementary Fig. 3c, d. Supernatant was discarded and the cell pellet was washed with 10 volumes of cold PBS and centrifuged at 380 rcf for 5 min. Supernatant was removed and the cell pellet was suspended in 10 volumes of Lysis Buffer (10 mM Hepes pH 7.9, 1.5 mM $MgCl_2$, 10 mM KCl, 0.5 mM DTT, 1.5 mM PMSF), sulfuric acid was added in a final concentration of 0.2M and the suspension was incubated on ice for 30 min before being centrifuged at 10,080 rcf for 10 min at 4°C. Next, the supernatant fraction was collected and TCA was added in a final concentration of 20%. Samples were vortexed and kept on ice for 1 h. Next, the samples were centrifuged for 15 min at 18,600 rcf at 4°C. The supernatant was discarded and the pellet was washed with 1 ml of ice cold (-20°C) acetone. After centrifugation at 18,600 rcf at 4°C for 5 min, acetone was removed carefully using a centrifugal evaporator and the pellet was resuspended in TE buffer and stored at -80°C.

Estimation of the proportion of the inhibited transcriptome

For calculating the percentage of the normally transcribed genome showing transcription inhibition previously published nRNA-seq data (NO UV and +UV 2h from[1]) were used to determine the actively transcribed regions where signal ratio (Log_2 FC (+UV/NOUV)) < 0. All active transcripts of length over 100kb were trimmed up to 100kb and were divided to genomic bins of 1kb. Read-depth normalized and exon-free nRNA-seq reads of each of the two conditions were counted on each genomic bin of each transcript, and for each of the n{1,2,...,100} bin positions the average Log_2 FC (+UV/NOUV)) ratio was calculated for the set of the active transcripts. This resulted to a vector of size 100, with Log_2 FC (+UV/NOUV)) values >= 1 for the first 28 bins, implying transcription clearance on the first 28kb of the active transcriptome upon UV damage, while for the last 72 bins Log_2 FC (+UV/NOUV)) values < 0, implying transcription inhibition upon UV damage. To calculate the total proportion of the active transcriptome where transcription was inhibited, the coverage (in bp) of all the normally actively transcribed elements (see above for definition of these loci) located within 28 kb from TSS were summed up and divided by the total length of all the actively transcribed elements, resulting to 63.65 %.

Reactome pathway analysis

DAR-gain regions were associated with active transcripts, either directly by searching for genomic overlaps with annotated promoters (this resulted to 2,767 DAR-gain at promoter) or by searching genomic overlaps with intergenic/intragenic FANTOM5 enhancers (this resulted to 820 DAR-gain at enhancers). DAR-gain at enhancers were functionally defined by analysing predicted enhancer-promoter associations retrieved from the FANTOM5 project site from 'enhancer.binf.ku.dk/presets/human.associations.hdr.txt.gz' and generated an additional 1,268 gene targets. A total of 3,284 of unique gene names were finally used to perform REACTOME pathway enrichment analysis using the R package ReactomePA: https://bioconductor.org/packages/release/bioc/html/ReactomePA.html.

Supplementary References

1. Lavigne, M. D., Konstantopoulos, D., Ntakou-Zamplara, K. Z., Liakos, A. & Fousteri, M. Global unleashing of transcription elongation waves in response to genotoxic stress restricts somatic mutation rate. *Nature Communications* **8**, 2076 (2017).
2. The FANTOM Consortium *et al.* An atlas of active enhancers across human cell types and tissues. *Nature* **507**, 455–461 (2014).
