# Supplementary material for: Continuous transcription initiation guarantees robust repair of all transcribed genes and regulatory regions
Source: Nat Commun. 2020 Feb 14;11:916. doi: 10.1038/s41467-020-14566-9 (PMC7021815; doi:10.1038/s41467-020-14566-9)
Supplement: Supplementary file 3 — Description of Additional Supplementary Files [file 41467_2020_14566_MOESM3_ESM.pdf]

**Description of Additional Supplementary Files**

File Name: Supplementary Data 1
Description: Lists of peaks/genes/coordinates of genomic regions used in the study
