# Supplementary material for: Continuous transcription initiation guarantees robust repair of all transcribed genes and regulatory regions
Source: Nat Commun. 2020 Feb 14;11:916. doi: 10.1038/s41467-020-14566-9 (PMC7021815; doi:10.1038/s41467-020-14566-9)
Supplement: Supplementary file 5 — Reporting Summary [file 41467_2020_14566_MOESM5_ESM.pdf]

nature research

Corresponding author(s): Matthieu D. Lavigne
Maria Fousteri

Last updated by author(s): Dec 18, 2019

Reporting Summary

Nature Research wishes to improve the reproducibility of the work that we publish. This form provides structure for consistency and transparency in reporting. For further information on Nature Research policies, see Authors & Referees and the Editorial Policy Checklist.

Statistics

For all statistical analyses, confirm that the following items are present in the figure legend, table legend, main text, or Methods section.

n/a	Confirmed	
☒	☐	The exact sample size (*n*) for each experimental group/condition, given as a discrete number and unit of measurement
☐	☒	A statement on whether measurements were taken from distinct samples or whether the same sample was measured repeatedly
☐	☒	The statistical test(s) used AND whether they are one- or two-sided<br>*Only common tests should be described solely by name; describe more complex techniques in the Methods section.*
☒	☐	A description of all covariates tested
☒	☐	A description of any assumptions or corrections, such as tests of normality and adjustment for multiple comparisons
☒	☐	A full description of the statistical parameters including central tendency (e.g. means) or other basic estimates (e.g. regression coefficient) AND variation (e.g. standard deviation) or associated estimates of uncertainty (e.g. confidence intervals)
☐	☒	For null hypothesis testing, the test statistic (e.g. *F*, *t*, *r*) with confidence intervals, effect sizes, degrees of freedom and *P* value noted<br>*Give P values as exact values whenever suitable.*
☒	☐	For Bayesian analysis, information on the choice of priors and Markov chain Monte Carlo settings
☒	☐	For hierarchical and complex designs, identification of the appropriate level for tests and full reporting of outcomes
☐	☒	Estimates of effect sizes (e.g. Cohen's *d*, Pearson's *r*), indicating how they were calculated

Our web collection on statistics for biologists contains articles on many of the points above.

Software and code

Policy information about availability of computer code

Data collection

To retrieve the in-house next generation sequencing raw files (FASTQ) from the EMBL Genomics Core Facilities servers, Aspera connect version 3.9.6 was used. To retrieve external sra files, sra toolkit version 2.9.6 was used.

Data analysis

To perform quality control on the FASTQ files FastQC version 0.11.5 was used. To perfom adapter clipping and qulaity trimming on the sequenced reads cutadapt version 2.4 was used, with a quality threshold of 20 ascii(phred quality + 33) and minimum read length of 10 bp. Nascent RNA-seq FASTQ files were aligned to the human reference genome using bowtie bowtie version 1.2, while ChIP-seq datasets by using bwa vesrion 0.7.12. Aligned reads were further processed using samtools version 1.9 to generate bam files and to retrieve only high quality alignments. To generate genome coverage files, bedtools version 2.25.0 was used. BigWig files compatible with genome browsers were created using ucsc tools version 370. To perform peak calling on the ATAC-seq alignments, MACS2 version 2.1.1 was used. To maximize the sensitivity of the detection of open chromatin regions. In particular, ---nomodel --shift 100 --extsize 200, --broad --shift 100 --extsize 200, --nomodel --shift 37 --extsize 73, --broad --shift 37 --extsize 73, --broad --nomodel --shift 37 --extsize 73 --keep-dup all, --broad --nomodel --shift 100 --extsize 200 --keep-dup all and --nomodel --shift 75 --extsize 150 --keep-dup all runs were combined, and detected peaks were filtered using fdr < 0.05 and fold change > 1. Only peaks present in 5 out of 7 methods were kept for further analysis. To perform peak calling on the ChIP-seq alignments, SICER version 1.1 was used. To perform summit peak calling on the CAGE-seq alignments, a python custom algorithm was implemented and applied. Briefly, for any active genomic element of interest, the 2kb flanks around the center of the region were devided to 5 bp genomic segment, and sense CAGE-seq signal was counted to retrieve the most enriched segment. Each of these values were then compared to the the putative summit background distribution coming from a set of inactive genomic element. All putative summits with a value over the mean of this distribution, were characterized as CAGE summits. Heamaps of NGS signal on genomic regions of interest were generated using seqMINER version 1.3.3. Custom code was implemented using R version 3.5, python version 3, perl and bash. Pathway enrichment analysis was performed using Reactome database.

Average profiles of NGS alignments: For each set of genomic elements of interest, alignment density matrices were generated using the seqMINER tool. These matrices were imported to R scripts to generate average profiles using ggplot2 package.

Genomic regions annotation pipeline: All genomic elements of interest were characterized as active, inactive and repressed, using a

custom pipeline. To annotate Transcription Start Sites (TSSs), all known protein coding and non-coding RNA hg19 RefSeq transcripts release 86 were downloaded from UCSC table browser (http://genome-euro.ucsc.edu/cgi-bin/hgTables). For each transcript, a biotype was assigned using BioMart (www.biomart.org), and all the small non-coding RNAs were excluded. For all the gene models containing multiple alternative transcripts, TSS neighborhoods of a 100 bp window were clustered together, and only the longest transcript was kept, resulting to 30,473 transcripts. Transcripts were then separated into 3 groups, based on their transcriptional activity. TSS coordinates were extended to 2kb on each direction and were tested for overlap with the Pol II-ser2P -UV, H3K27ac -UV and H3K27me3 -UV peak sets. Regions overlapping with an Pol II-ser2P -UV and H3K27ac -UV peak, were characterized as active, those overlapping with an H3K27me3 -UV peak, but not with an Pol II-ser2P -UV neither with a H3K27ac -UV peak were characterized as repressed, and those that did not overlap with any of the above peak sets were characterized as inactive. Any region overlapping with both H3K27ac -UV and H3K27me3 -UV peaks were excluded from the rest of the analysis. This resulted to 15,819 active, 2,943 repressed and 7,608 inactive transcripts. To further classify the active TSSs in terms of transcription directionality, the annotation was split up into unidirectional and bidirectional references. All active transcript pairs with opposite direction of transcription, where -2 kb ≤ TSSdistance ≤ +2 kb, TSSdistance = TSS coordinate_forward strand –TSScoordinate reverse strand (interCAGE distance) were characterized as bidirectional, while the rest of the annotations were characterized as unidirectional. Bidirectional pairs were further categorized into two groups of annotations: convergent bidirectional transcript pairs with TSSdistance ≤ 100 bp, and divergent bidirectional transcript pairs with TSSdistance > 100 bp. To optimize the categorization of convergent and divergent transcript pairs, TSS coordinates were redefined by scanning in a radius of 250 bp, to detect the nucleotide occupied by the maximum sense CAGE signal. Any bidirectional pair with a non-significant CAGE peak in the aforementioned region was excluded from the analysis. This finally resulted to 12,859 unidirectional transcripts and 2,822 active bidirectional TSS pairs, 1,806 of which were characterized as divergent and 1,016 as convergent.
To annotate upstream antisense (uaRNA) and downstream antisense (daRNA) transcripts (referred as an ensemble to PROMPTs in this paper for convenience). Only the active unidirectional mRNA TSSs were used. For all the genes annotated with more than one mRNA transcript, only the leftmost TSS (for + strand genes), and rightmost TSS (for - strand genes) were considered for the rest of the analysis. The antisense CAGE peak with the highest summit in the region ranged from -2 kb upstream to +1 kb downstream of each unidirectional TSS was considered to be the main PROMPT TSS for further analyses. The above procedure was also repeated for the inactive transcript set, to estimate the highest CAGE summit background distribution. The putative active PROMPT CAGE summits, which were higher than the average of the summit background distribution, were considered as active. This resulted to 5,366 pairs of active unidirectional - PROMPT TSSs, which were categorized to 1,444 divergent and 3,922 convergent pairs, as described above. By focusing on the divergent loci, the dynamics of transcription could be studied at play in each direction, without having to deal with interference from either direction.
To annotate enhancer Transcription Start Sites (eTSSs), all 65,423 human enhancers from phases 1 and 2 of the FANTOM5 project from http://fantom.gsc.riken.jp/ 5/datafiles/phase2.2/extra/Enhancers/human_permissive_enhancers_phase_1_and_2.bed.gz, and the center of each annotation was considered as the corresponding transcription start site. Enhancers were separated to 6,766 active, 4,730 repressed and 39,227 inactive following the pipeline described above. Active intergenic enhancers were further analyzed, and all the eTSSs within a distance of 10kb from nearby active transcripts, or neighbor eTSSs within a distance of 2kb were excluded. The rest of the intergenic eTSSs were extended to 1kb in both directions, and sense and antisense maximum CAGE summit heights were detected for each reference. This procedure was also repeated for the inactive enhancer set and inactive sense and antisense highest CAGE summit background distributions were estimated as described above. Finally, the putative active intergenic sense and antisense CAGE summits which were higher the averages of the summit background distributions, were considered as active. This resulted to 1,228 active intergenic eTSSs.

EI calculation pipeline: Promoter escape analysis was performed for a subset of active unidirectional and bidirectional transcripts, PROMPTs and active enhancers. In particular, to avoid the inclusion of Pol II-ser2P reads mapped in overlapping promoters and gene bodies, only active divergent unidirectional transcript - PROMPT pairs were considered, where TSSdistance > 100 bp, TSSdistance= TSS coordinate forward reference-TSS coordinate reverse reference, active divergent bidirectional transcript pairs with TSSdistance > 100 bp and active intergenic enhancers with no nearby transcripts within 10 kb and no nearby eTSSs within 2kb. For TSSs and PROMPT-TSSs promoter Escape Indexes (EI) were calculated as previously defined28, by taking the average coverage in rpm in the gene body (density in gene body was abbreviated as Db and ranged from 101 bp to 2 kb downstream of TSS or 101 bp downstream of TSS to TTS for genes larger or smaller than 2 kb respectively) divided by the average coverage on the promoter-proximal region (Dp) ranged from 250 bp upstream to 100 bp downstream of TSS. For enhancer escape analysis, EI was calculated as above, where Density of reads at enhancer flanks (Df) is calculated for the regions ranging from -2 kb to -100 bp upstream of eTSS and from +100 bp to +2 kb downstream of eTSS, while density of reads on enhancer TSS (De) is calculated for the regions ranging from 100 bp upstream to 100 bp downstream of eTSS.

Proportion of Inhibited transcriptome estimation pipeline: For calculating the percentage of the normally transcribed genome showing transcription inhibition previously published nRNA-seq data (NO UV and +UV 2h from28) were used to determine the actively transcribed regions where signal ratio (Log2 FC (+UV/NOUV)) < 0. All active transcripts of length over 100kb were trimmed up to 100kb and were divided to genomic bins of 1kb. Read-depth normalized and exon-free nRNA-seq reads of each of the two conditions were counted on each genomic bin of each transcript, and for each of the n{1,2,...,100} bin positions the average Log2 FC (+UV/NOUV)) ratio was calculated for the set of the active transcripts. This resulted to a vector of size 100, with Log2 FC (+UV/NOUV)) values >= 1 for the first 28 bins, implying transcription clearance on the first 28kb of the active transcriptome upon UV damage, while for the last 72 bins Log2 FC (+UV/NOUV)) values < 0, implying transcription inhibition upon UV damage. To calculate the total proportion of the active transcriptome where transcription was inhibited, the coverage (in bp) of all the normally actively transcribed elements (see above for definition of these loci) located within 28 kb from TSS were summed up and divided by the total length of all the actively transcribed elements, resulting to 63.65 %

For manuscripts utilizing custom algorithms or software that are central to the research but not yet described in published literature, software must be made available to editors/reviewers. We strongly encourage code deposition in a community repository (e.g. GitHub). See the Nature Research guidelines for submitting code & software for further information.

Data

Policy information about availability of data

All manuscripts must include a data availability statement. This statement should provide the following information, where applicable:

- Accession codes, unique identifiers, or web links for publicly available datasets
- A list of figures that have associated raw data
- A description of any restrictions on data availability

The data reported in this manuscript have been deposited with the Gene Expression Omnibus under accession code GSE125181

Field-specific reporting

Please select the one below that is the best fit for your research. If you are not sure, read the appropriate sections before making your selection.

☒ Life sciences ☐ Behavioural & social sciences ☐ Ecological, evolutionary & environmental sciences

For a reference copy of the document with all sections, see nature.com/documents/nr-reporting-summary-flat.pdf

Life sciences study design

All studies must disclose on these points even when the disclosure is negative.

Sample size	Statistical methods o predetermined sample size were not applied.
Data exclusions	There were no data exclusions in this study.
Replication	For ATAC-seq and ChIP-seq replicated datasets, PCC correlation coefficient was calculated on summarized read densities on peak regions.
Randomization	There was no randomization of experiments.
Blinding	N/A

Reporting for specific materials, systems and methods

We require information from authors about some types of materials, experimental systems and methods used in many studies. Here, indicate whether each material, system or method listed is relevant to your study. If you are not sure if a list item applies to your research, read the appropriate section before selecting a response.

Materials & experimental systems

n/a	Involved in the study	
☐	☒	Antibodies
☐	☒	Eukaryotic cell lines
☒	☐	Palaeontology
☒	☐	Animals and other organisms
☒	☐	Human research participants
☒	☐	Clinical data

Methods

n/a	Involved in the study	
☐	☒	ChIP-seq
☒	☐	Flow cytometry
☒	☐	MRI-based neuroimaging

Antibodies

Antibodies used	All antibodies used in this study are reported in the ChIP-seq section.
Validation	See manufacturer's webpage for more information.

Eukaryotic cell lines

Policy information about cell lines

Cell line source(s)	VH10 htert immortalized human skin fibroblasts (Check also methods "cell culture and treatment" section)
Authentication	See above
Mycoplasma contamination	The cell lines used in this study have been tested negative for mycoplasma contamination.

Commonly misidentified lines
(See ICLAC register)

No commonly misidentified cell lines were used for this study

ChIP-seq

Data deposition

☒ Confirm that both raw and final processed data have been deposited in a public database such as GEO.

☒ Confirm that you have deposited or provided access to graph files (e.g. BED files) for the called peaks.

Data access links
May remain private before publication.

The data reported in this manuscript have been deposited with the Gene Expression Omnibus under accession code GSE125181

Files in database submission

All datasets included in Series GSE125181 are listed below:

GSM3564706 ATAC_NO_UV_1: ATAC-seq NOUV, replicate 1
GSM3564707 ATAC_NO_UV_2: ATAC-seq NOUV, replicate 2
GSM3564708 ATAC_+UV_1: ATAC-seq +UV, replicate 1
GSM3564709 ATAC_+UV_2: ATAC-seq +UV, replicate 2
GSM3564710 H3K27ac_NO_UV_1: H3K27ac ChIP-seq NOUV, replicate 1
GSM3564711 H3K27ac_NO_UV_2: H3K27ac ChIP-seq NOUV, replicate 2
GSM3564712 H3K27ac_+UV_1: H3K27ac ChIP-seq +UV, replicate 1
GSM3564713 H3K27ac_+UV_2: H3K27ac ChIP-seq +UV, replicate 2
GSM3564714 Input_NO_UV: Input dataset
GSM3564715 H3K27me3_NO_UV: H3K27me3 ChIP-seq -UV
GSM3564716 H3K27me3_+UV: H3K27me3 ChIP-seq +UV
GSM3564717 RNAPII-hypo_+UV_+DRB_X4h: RNAPII hypo ChIP-seq +UV +DRB X4h
GSM3564718 RNAPII-hypo +UV_-DRB_X4h: RNAPII hypo ChIP-seq +UV -DRB X4h
GSM3564719 RNAPII-hypo_NO UV_+DRB_X4h: RNAPII hypo ChIP-seq NOUV +DRB X4h
GSM3564720 RNAPII-hypo_+UV_X2h: RNAPII hypo ChIP-seq +UV X4h

Genome browser session
(e.g. UCSC)

Available upon request

Methodology

Replicates

ATAC-seq experiments and H3K27ac ChIP-seq experiments were performed in duplicates.

Sequencing depth

All experiments are single ended.

ATAC-seq NOUV, replicate 1 : library depth = 57973024, read length = 50 bps
ATAC-seq NOUV, replicate 2 : library depth = 47008087, read length = 50 bps
ATAC-seq +UV, replicate 1 : library depth = 52518646, read length = 50 bps
ATAC-seq +UV, replicate 2 : library depth = 50503219, read length = 50 bps

H3K27ac NOUV replicate 1 : library depth = 36764915 reads, read length = 51 bps
H3K27ac NOUV replicate 2 : library depth = 20072220 reads, read length = 51 bps
H3K27ac +UV replicate 1 : library depth = 43859328 reads, read length = 51 bps
H3K27ac +UV replicate 2 : library depth = 21817328 reads, read length = 51 bps
INPUT : library depth = 56346145 reads, read length = 51 bps

H3K27me3 NOUV : library depth = 22470998, read length = 51 bps
H3K27me3 +UV : library depth = 30152268, read length = 51 bps

RNAPII hypo ChIP-seq +UV +DRB X4h : library depth = 36079747, read length = 51 bps
RNAPII hypo ChIP-seq +UV -DRB X4h : library depth = 32895923, read length = 51 bps
RNAPII hypo ChIP-seq NOUV +DRB X4h : library depth = 41742983, read length = 51 bps
RNAPII hypo ChIP-seq +UV X2h : library depth = 36835670, read length = 51 bps

Antibodies

anti-H3K27ac (ab4729, Abcam), H3K27me3 (07-449, Millipore), anti-8WG16 (Pol II-hypo) (05-952, Millipore)

Peak calling parameters

To call ATAC-seq peaks, in order to increase the sensitivity of the calls, a combination of MACS2 runs was applied with parameters:

-f BED -g hs --nomodel --shift 100 --extsize 200
-f BED -g hs --broad --shift 100 --extsize 200
-f BED -g hs --nomodel --shift 37 --extsize 73
-f BED -g hs --broad --shift 37 --extsize 73
-f BED -g hs --broad --nomodel --shift 37 --extsize 73 --keep-dup all
-f BED -g hs --broad --nomodel --shift 100 --extsize 200 --keep-dup all
-f BED -g hs --nomodel --shift 75 --extsize 150 --keep-dup all

ChIP-seq peak calling was applied using SICER version 1.1 with control datasets, using parameters:

window = 200 and gap = 1

Data quality

FastQC was used to perform quality controls on the raw FASTQ files of NGS data.

Peaks with FDR less than 5% and fold enrichment over 5-fold:

ATAC-seq NOUV rep 1 : 90341
ATAC-seq NOUV rep 2 : 75644
ATAC-seq +UV rep 1 : 115292
ATAC-seq +UV rep 2 : 133336

H3K27ac NOUVreplicate 1 : 15503
H3K27ac NOUV replicate 2 : 17077
H3K27ac +UV replicate 1 : 8935
H3K27ac +UV replicate 2 : 19265

H3K27me3 NOUV: 7301
H3K27me3 +UV: 6328

Software

All software used to analyze ChIP-seq data is described in the data collection section.
